# Changes of macular vessel density and thickness in gas and silicone oil tamponades after vitrectomy for macula-on rhegmatogenous retinal detachment

**DOI:** 10.1186/s12886-021-02160-6

**Published:** 2021-11-15

**Authors:** Yang Liu, Boya Lei, Rui Jiang, Xin Huang, Min Zhou, Gezhi Xu

**Affiliations:** 1 Department of Ophthalmology, Eye and ENT Hospital of Fudan University, No.83, Fen Yang Road, Shanghai, 200031 China; 2 Shanghai Key Laboratory of Visual Impairment and Restoration, Fudan University, Shanghai, China; 3 Key Laboratory of Myopia of State Health Ministry, Fudan University, Shanghai, China

**Keywords:** Macular vessel density, Retinal thickness, Rhegmatogenous retinal detachment, Silicone oil tamponade, Gas tamponade

## Abstract

**Purpose:**

To investigate the macular vessel density and thickness in macular-on rhegmatogenous retinal detachment (RRD) after vitrectomy with gas and silicone oil (SO) tamponade.

**Methods:**

Patients with macular-on RRD eyes, treated with a single successful vitrectomy with gas or SO tamponade and a minimum 30 months follow-up, were reviewed. Best-corrected visual acuity (BCVA), macular vessel density and retinal thickness by using optical coherence tomography angiography, were compared to the contralateral eyes.

**Results:**

Sixteen eyes with gas tamponade and 17 eyes with SO tamponade were included in the study. LogMAR best-corrected visual acuity (BCVA) slightly improved from 0.25 ± 0.18 (Snellen 20/36) to 0.17 ± 0.23 (Snellen 20/30) in eyes with gas tamponade, and decreased from 0.30 ± 0.22 (Snellen 20/40) to 0.49 ± 0.28 (Snellen 20/62) in eyes with SO tamponade. The parafoveal vessel densities in superficial vascular complex (SVC) and the corresponding inner retinal thickness (IRT) were similar between the affected eyes and the contralateral eyes in gas tamponade group (*P* = 0.578, *P* = 0.943), while significantly reduced in the affected eyes, compared to the contralateral eyes in SO tamponade group (*P* < 0.001, *P* < 0.001).

**Conclusion:**

Eyes in SO tamponade group had worse BCVA, lower SVC vessel densities and thinner corresponding IRT after vitrectomy for macular-on RRD, than those in gas tamponade group.

## Introduction

Rhegmatogenous retinal detachment (RRD) is characterized by a full-thickness break in the neurosensory retina (NSR), variable degrees of vitreous traction, and the passage of liquefied vitreous through the break into the subretinal space. RRD requires appropriate surgery to halt its progression to prevent further damage to vision. Pars plana vitrectomy (PPV) plus endotamponade is a frequent surgical option for RRD treatment. The preferred tamponades include expandable gas or silicone oil (SO) as the high surface tension and expansivity can help support the adhesions of the NSR [1, 2].

Successful retinal attachment can be achieved with a tamponade; however, the SO tamponade has a reported association with unexplained vision loss [3–10]. By contrast, no similar side effects have been found in eyes with expandable gas tamponade [8]. Since the first report of a poorly recognized deterioration in visual acuity after SO tamponade, an increasing number of studies have focused on SO-related vision loss. In addition to loss of visual acuity, a central scotoma has been a cardinal complaint. For example, Newsom et al. were the first to objectively assess diffuse central scotoma in patients after the SO tamponade [3], and their findings were subsequently supported by the studies by Shalchi et al. and Herbert et al. [6, 11] A central visual dysfunction suggested a macula-limited origin, and the anatomic and electrophysiological results also provided detailed evidence to confirm a macular involvement in the pathogenetic process [4–7, 12].

Macular architectural changes can be detected by optical coherence tomography (OCT), while optical coherence tomography angiography (OCTA) can provide vessel details in addition to the macular retinal anatomy. Fluorangiography, thanks to the use of fluorescein, is capable of evaluating vascular hydrodynamics. OCT-A, even though it is not a dynamic evaluation (as fluorescein angiography), is able to detect vessel architecture thus evaluate the perfusion function of macular vessels. The normal scan mode can precisely reflect surface or deep capillary flow densities on the macula and precisely capture the changes in the foveal avascular zone (FAZ) [13, 14].

The aim of the present study was therefore to obtain OCTA and OCT findings in patients with macula-on primary RRD who had undergone PPV treatments, and to compare the differences in macular microvascular blood supply and thickness in cohorts administered with expandable gas versus SO tamponades. Our hypothesis was that these findings could help to account for the oddity observed in patient responses to these two tamponades.

## Methods

### Participants and examinations

This was a single-centered, retrospective cohort study. The medical records were reviewed for consecutively patients who had undergone vitrectomy for a primary macular-on RRD with a gas (octafluoropropane, C3F8) or SO tamponade between July 2015 and June 2017 at the Department of Ophthalmology, Eye and ENT Hospital of Fudan University.

The surgical procedures were performed with standard 23-gauge, three-port par plana vitrectomy. The central core vitreous was removed at first. Triamcinolone acetonide aqueous suspension was then applied to visualize the posterior hyaloid. The posterior vitreous and peripheral vitreous were carefully removed in sequence. Perfluorocarbon liquid was used to stabilize the floating retina if necessary. Drainage of subretinal fluid was performed with an aspiration needle under the help of perfluorocarbon liquid, air infusion, or both. After retina was internally reattached, retinal breaks were treated with laser photocoagulation through an endoprobe, with two to three rows of burns placed around each break. After air–fluid exchange, gas or SO tamponade was performed, and patients were asked to stay in a specific position according to the location of the retinal breaks.

The choice of intraocular tamponade depended on the surgeons’ discretion, basically based on the degree of proliferative vitreoretinopathy (PVR) and the locations of retinal breaks. The PVR classification was based on The updated Retina Society Classification (1991) as previously described [15]. Patients with persistent retinal attachment of the affected eyes for at least 30 months after vitrectomy and with healthy contralateral eyes were included in the study. Eyes with SO tamponade would be observed for at least 3 months after vitrectomy; when attached and stable retina was observed at 3 months after vitrectomy, the patient will register and queue up for silicone oil removal. When SO-related uncontrolled elevated IOP occurred, SO removal will be arranged in advance. Silicone oil had been removed in all eyes treated with a SO tamponade when the patients were included in the study.

Patients with any of the following characteristics were excluded: 1) high myopia with axial length over 26.5 mm in any eye, 2) diabetes mellitus or other diseases affecting retinal blood flow, 3) anisometropia between the affected eye and contralateral eye (the difference in axial length of both eyes is more than 0.5 mm), 4) preexisting ocular diseases or history of ocular trauma or surgeries, except for cataract surgery and 5) additional surgical procedures except for posterior vitreous detachment induction and posterior vitreous cortical excision were performed in macular area during vitrectomy, for example internal limiting membrane peeling.

The medical records before surgery were reviewed for both eyes of all patients. The data included best-corrected visual acuity (BCVA; in Snellen visual acuity ratios, and in logarithms of the minimum angle of resolution [logMAR] when used for the purpose of statistical analysis), intraocular pressure (IOP), axial length, and duration between symptoms and surgery.

During the follow-up, both eyes in patients with attached retinas underwent a complete ophthalmic examination, including fundus examination, BCVA, IOP, OCTA and OCT scans. The patient’s blood pressure was recorded at the time of the OCTA scans, and the mean arterial pressure (MAP) was calculated as the diastolic blood pressure plus one-third of the difference between the diastolic blood pressure and the systolic blood pressure. The ocular perfusion pressure (OPP) was calculated by subtracting the IOP from two-thirds of the MAP [16]. An IOP of over 21 mmHg was defined as IOP elevation.

This study was conducted in accordance with the tenets of the Declaration of Helsinki and was approved by the Institutional Review Board of the Eye and ENT Hospital of Fudan University.

### OCTA and OCT acquisitions

The OCTA and OCT scans were acquired using a spectral domain OCT system (software Version 2017.1.0.155; RTVue-XR Avanti, Optovue Inc., Fremont, CA). Three dimensional (3D) OCTA scans were acquired over 3 × 3 mm and 6 × 6 mm areas of the macula. The image quality of all OCTA scans was assessed according to the quality index (QI) [17]. Images with QI < 6 were excluded from the analysis. Two OCT-A scans were obtained for both eyes by a single operator during the same visit, and the average of the two measurements was calculated for statistical analysis. OCT scans were acquired for retinal thickness analysis with a retinal map protocol. The segmentation of OCT and OCTA scans were checked before analysis to avoid automatic errors. Both eyes of each participant were examined and scanned at the same visit.

### OCTA measurements

An en-face retinal angiogram was generated by the RTVue-XR Avanti software, which automatically identified the projection signals from the internal limiting membrane (ILM) to the retinal pigment epithelium (RPE). Vessel density (VD) was defined as the percentage of the area occupied by vessels within the segmented area [18]. All measurements were acquired using the built-in RTVue-XR Avanti software.

The VDs in the macular areas were measured from a 6 × 6 mm macular cube. The parafoveal area was defined as an annulus centered at the fovea with an inner diameter of 1 mm and an outer diameter of 3 mm. The superficial vascular complex (SVC) and deep vascular complex (DVC) were analyzed separately in the macular VD analysis. The predefined boundaries provided by the Optovue software were used for the SVC and the DVC analysis. The SVC was located between the inner limiting membrane (ILM) and 10 μm above the inner plexiform layer and the inner nuclear layer (IPL-INL) junction, while the DVC was located between 10 μm above the IPL-INL junction and 10 μm below the outer plexiform layer and outer nuclear layer (OPL-ONL) junction. The foveal avascular zone (FAZ) was measured using the software with a 3 × 3 mm macular cube. The retinal en face slab (the retina from the ILM to 10 μm below OPL-ONL junction) was used for the evaluation of the FAZ.

### Retinal thickness analyses

Retinal thickness measurements were calculated within the parafoveal area as described before using the retinal map protocol with the built-in Avanti RTVue-XR software, as previously reported [19]. The full, inner, and outer retinal layer thicknesses (FRT, IRT, ORT) were calculated as the distance between ILM and the middle of RPE, the ILM and IPL-INL junction, and the IPL-INL junction and the middle of RPE, respectively.

### Statistical analysis

Data are presented as the mean ± standard deviation. The paired-t test was used to compare the changes in the clinical variables over time or to compare clinical data between both eyes of the patient. The Mann–Whitney U test or Fisher-Chi Square were used to compare the clinical variables in different groups. Values of *P* < 0.05 were considered statistically significant. Statistical analyses were performed using SPSS for Windows Version 17.0 (SPSS Inc., Chicago, IL, USA).

## Results

### Basic information

In total, 33 patients (11 males and 22 females) with an average age of 53.5 ± 10.0 years (from 33 to 66 years) were enrolled in this study. They complained of decreased vision or visual-field defects for 22.0 ± 17.7 days (from 3 to 60 days). The mean axial length was 24.00 ± 0.97 mm (from 22.42 to 26.39 mm) in the affected eyes. The mean follow-up duration was 36.1 ± 3.6 months (from 30 to 43 months). The mean logMAR BCVA was 0.28 ± 0.20 (Snellen equivalent 20/38, from 0.00 to 0.70) at presentation and 0.33 ± 0.30 (Snellen equivalent 20/43, from 0.00 to 1.00) at the last follow-up (*P* = 0.348, Paired-t test). The mean IOP was 13.1 ± 2.6 mmHg (from 9.50 to 19.3 mmHg) at presentation and 17.6 ± 4.1 mmHg (from 11.3 to 27.0 mmHg) at the last follow-up (*P* < 0.001, Paired-t test).

Of the affected eyes, 16 eyes were treated with a gas tamponade and the other 17 eyes were treated with a SO tamponade. The PVR grades were similar in both groups (*P* = 1.000, Fisher-Chi Square test). The extension of RRD (number of clock-hours involved) and number of retinal tears or breaks were similar in both groups (*P* = 0.908, *P* = 0.685). The frequency of retinal breaks involving the inferior 4 clock-hours’ area was higher in SO tamponade group than in gas tamponade group (4/17 eyes versus 1/16 eyes), while the difference was not statistically significant (*P* = 0.335, Fisher-Chi Square test). The mean duration between surgery and silicone oil removal was 5.8 ± 2.3 months (from 2.3 to 13.2 months). The clinical characteristics of the affected eyes are shown in Table 1.

**Table 1:** Clinical characteristics of the affected eyes

	Eyes with gas tamponade	Eyes with silicone oil tamponade	*P* value (Mann-Whitney/Fisher-Chi Square test)
Age, years	55.5 ± 9.68	51.5 ± 10.1	0.159
Sex	4 M/12F	7 M/10F	0.465
Laterally	12R/4 L	9R/8 L	0.282
Duration between symptoms and surgery, days	18.2 ± 15.9	25.8 ± 19.0	0.168
Proliferative vitreoretinopathy grades, eyes	PVR A&B:8 PVR C:8	PVR A&B:8 PVR C:9	1.000
Extension of RRD (number of clock-hours involved)	3.7 ± 0.9	3.7 ± 0.7	0.908
Number of retinal tears/breaks	1.1 ± 0.3	1.2 ± 0.4	0.685
Frequency of retinal breaks involving the inferior 4 clock-hours’ area	1/17	4/16	0.335
Axial length, mm	23.9 ± 0.8	24.1 ± 1.1	0.792
Follow-up duration, months	36.3 ± 3.7	35.8 ± 3.7	0.698
LogMAR BCVA at presentation, Snellen equivalent	0.25 ± 0.18 20/36	0.30 ± 0.22 20/40	0.509
IOP at presentation, mmHg	12.5 ± 2.6	13.6 ± 2.5	0.171
LogMAR BCVA at the follow-up, Snellen equivalent	0.17 ± 0.23 20/30	0.49 ± 0.28 20/62	**< 0.001**
IOP at the follow-up	16.5 ± 3.2	18.6 ± 4.7	0.191

*PVR* Proliferative vitreoretinopathy, *RRD* Rhegmatogenous retinal detachment, *logMAR* Logarithm of the minimum angle of resolution, *BCVA* Best corrected visual acuity, *IOP* Intraocular pressure

LogMAR BCVA in eyes tamponaded with gas slightly improved after surgery (*P* = 0.300, Paired-t test), while the value in eyes treated with SO tamponade clearly decreased (*P* = 0.034, Paired-t test). No IOP elevation was found in the gas group during the follow-up, compared with the fellow eyes. In the SO group, five of the 17 eyes (29.4%) complained of a history of IOP elevation and all received medicine to lower the IOP. At the final visit, IOP normalized in 3 out of these 5 patients, while the remanent showed IOP elevation. Besides, four more eyes (23.5%) presented with IOP elevation at the final visit, without the detection of symptoms before and following treatment. In conclusion, nine eyes (52.9%) experienced with IOP elevation during follow up.

### Comparison of vessel density and retinal thickness between both eyes in patients receiving the gas tamponade

**Fig. 1:**
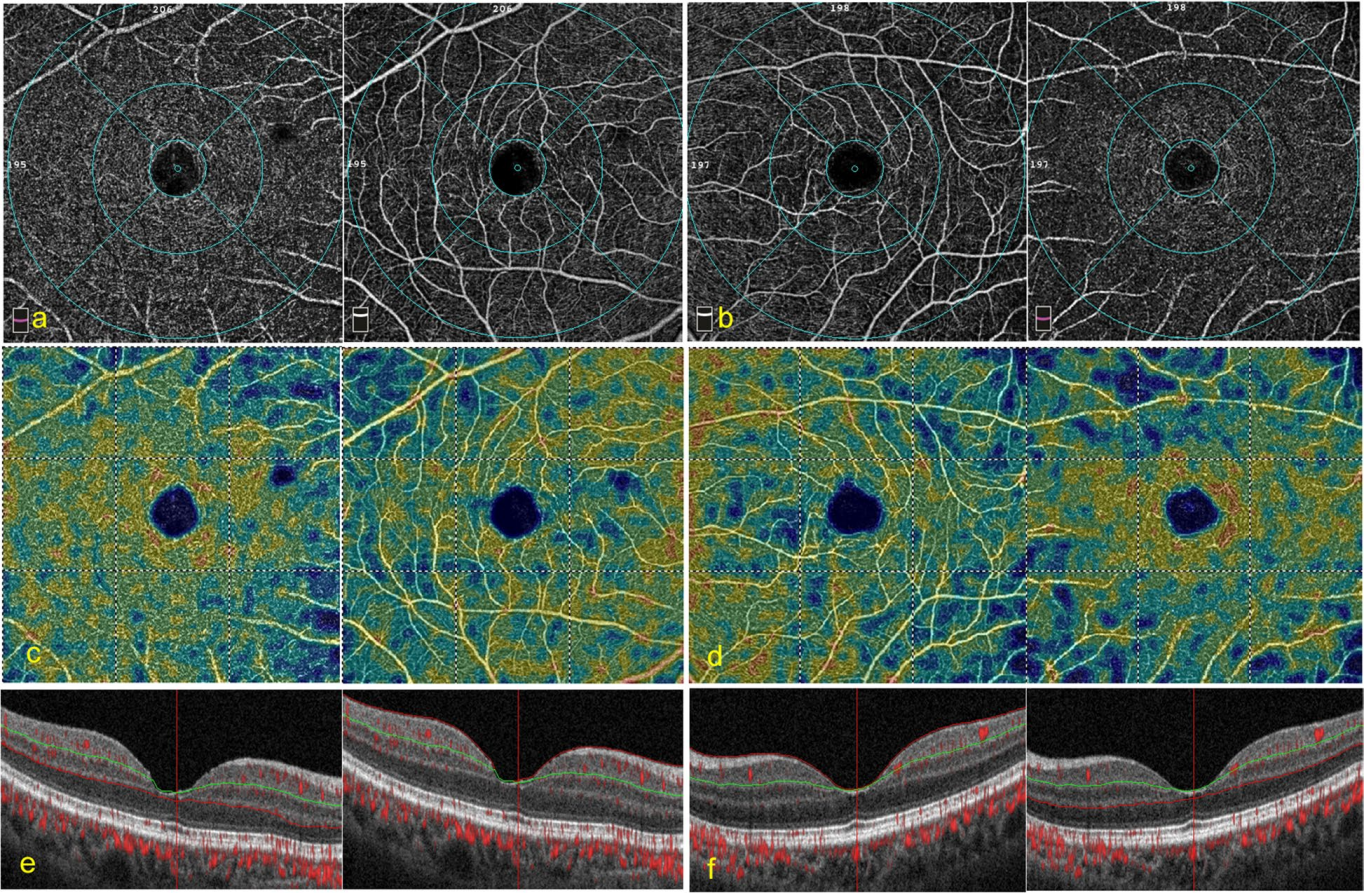
A representative case of OCTA in gas tamponade eyes. A 59-year-old female patient with macula-on retinal detachment (RD) in her right eye achieved reattachment in one shot. The preoperative best-corrected visual acuity (BCVA) was LogMAR 0.3 (Snellen equivalent 20/40). The intraocular pressure (IOP) was 10 mmHg. Postoperatively, BCVA increased to LogMAR 0.1 (Snellen equivalent 20/25), the IOP was 14 mmHg, with no history of IOP elevation. No significant difference of vessel density (VD) was found in the affected and contralateral eyes. **a** the 6 × 6 mm en face angiogram in the affected eye: left: the deep vessel complex (DVC); right: the superficial vessel complex (SVC); **b** the 6 × 6 mm en face angiogram in the contralateral eye: left: the SVC; right: the DVC; **c** the 6 × 6 mm false coloring angiogram in the affected eye: left: the DVC; right: the SVC; **d** the 6 × 6 mm false coloring angiogram in the contralateral eye: left: the SVC; right: the DVC; **e** the OCT B scan image with layered boundary details in the affected eye: left: the DVC; right: the SVC; **f** the OCT B scan image with layered boundary details in contralateral eye: left: the SVC; right: the DVC

For eyes treated with the gas tamponade, axial lengths were similar in the affected eyes and the contralateral healthy eyes. No statistically significant differences were found for FAZ, parafoveal VD (both SVC and DVC, Fig. 1), or retinal thickness (FRT, IRT, and ORT) between the affected and contralateral healthy eyes. The results are shown in Table 2.

**Table 2:** Comparison of vessel density and retinal thickness between both eyes in patients receiving the gas tamponade

	The affected eyes	The healthy eyes	*P* (Paired-t)
Axial length, mm	23.92 ± 0.75	24.11 ± 1.21	0.372
logMAR BCVA at presentation, Snellen equivalent	0.25 ± 0.18 20/36	0.07 ± 0.10 20/23	**0.001**
IOP at presentation, mmHg	12.5 ± 2.6	13.8 ± 2.8	**0.010**
logMAR BCVA at follow up, Snellen equivalent	0.17 ± 0.23 20/30	0.06 ± 0.09 20/23	**0.020**
IOP at follow up	16.5 ± 3.2	15.1 ± 2.0	0.103
Ocular perfusion pressure at follow up, mmHg	49.20 ± 6.41	50.61 ± 6.31	0.103
FAZ, mm^2^	0.304 ± 0.179	0.315 ± 0.111	0.693
Parafoveal VD of SVC (%)	44.13 ± 4.90	43.17 ± 4.30	0.578
Parafoveal VD of DVC (%)	50.24 ± 4.34	52.59 ± 4.36	0.120
Full retinal thickness, μm	319.13 ± 23.23	313.19 ± 11.17	0.294
Inner retinal thickness, μm	121.75 ± 15.97	122.06 ± 7.52	0.943
Outer retinal thickness, μm	195.44 ± 13.47	190.81 ± 8.26	0.075

*logMAR* Logarithm of the minimum angle of resolution, *BCVA* Best corrected visual acuity, *IOP* Intraocular pressure, *FAZ* Foveal avascular zone, *VD* Vessel density, *SVC* Superior vascular complex, *DVC* Deep vascular complex

### Comparison of vessel density and retinal thickness between both eyes in patients receiving SO tamponade

**Fig. 2:**
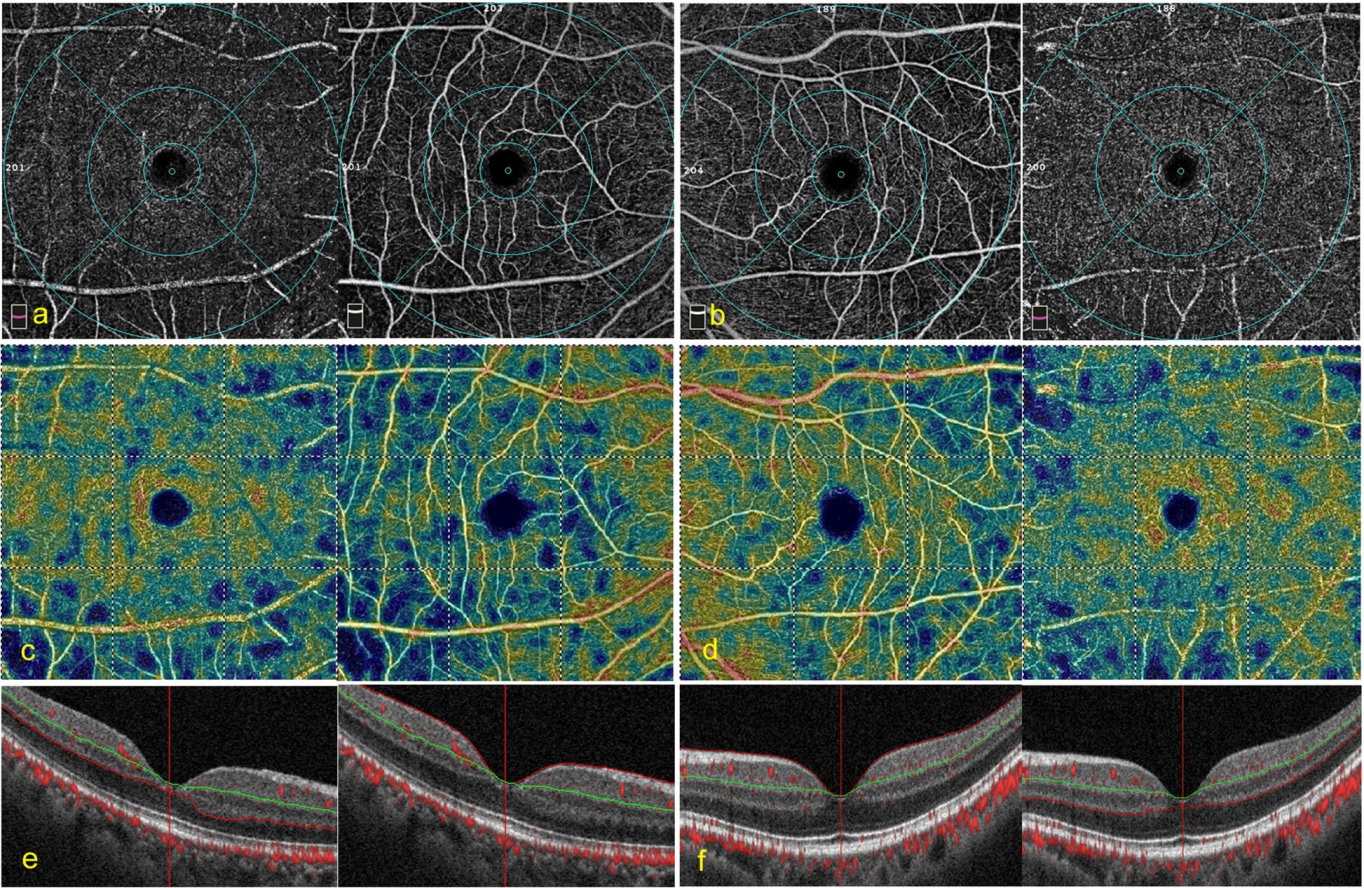
A representative case of OCTA in silicone oil (SO) tamponade eyes. A 53-year-old female patient with macula-on RRD in her right eye achieved reattachment in one shot. The period for SO in suit lasted for 4.5 months. The preoperative best-corrected visual acuity (BCVA) was LogMAR 0.22 (Snellen equivalent 20/33). The intraocular pressure (IOP) was 13 mmHg. Postoperatively, the BCVA decreased to LogMAR 0.52 (Snellen equivalent 20/67), the IOP was 19 mmHg. The patient complaint of a history of IOP elevation, with a limit high to 32 mmHg for 2 weeks. A significantly decreased parafoveal vessel density (VD) of superficial vessel complex was found in the affected eye, compared with the fellow eye. **a** the 6 × 6 mm en face angiogram in the affected eye: left: the deep vessel complex (DVC); right: the superficial vessel complex (SVC); **b** the 6 × 6 mm en face angiogram in the contralateral eye: left: the SVC; right: the DVC; **c** the 6 × 6 mm false coloring angiogram in the affected eye: left: the DVC; right: the SVC; **d** the 6 × 6 mm false coloring angiogram in the contralateral eye: left: the SVC; right: the DVC; **e** the OCT B scan image with layered boundary details in the affected eye: left: the DVC; right: the SVC; **f** the OCT B scan image with layered boundary details in the contralateral eye: left: the SVC; right: the DVC

In patients who undergone SO tamponade surgery, axial lengths were similar in the affected eyes and contralateral healthy eyes. IOP was higher and the ocular perfusion pressure was lower in the affected eyes during the follow-up, with statistically significant differences. No statistically significant differences were detected for FAZ, parafoveal VD of DVC, FRT, or ORT between the affected and contralateral healthy eyes. Compared to the healthy eyes, the parafoveal VD of the SVC (Fig. 2) and IRT were reduced in the affected eyes, and the differences were all statistically significant. The results are shown in Table 3.

**Table 3:** Comparison of vessel density and retinal thickness between both eyes in patients receiving the silicone oil tamponade

	Affected eye	Healthy eye	*P* (Paired-t)
Axial length, mm	24.1 ± 1.1	24.0 ± 1.1	0.199
logMAR BCVA at presentation, Snellen equivalent	0.30 ± 0.22 20/40	0.06 ± 0.15 20/23	**0.001**
IOP at presentation, mmHg	12.6 ± 2.5	15.5 ± 2.0	**0.001**
logMAR BCVA at follow up, Snellen equivalent	0.49 ± 0.28 20/62	0.02 ± 0.06 20/21	**< 0.001**
IOP at follow up	18.6 ± 4.7	16.4 ± 3.4	**0.006**
Ocular perfusion pressure at follow up, mmHg	41.0 ± 7.8	43.2 ± 6.8	**0.006**
FAZ, mm^2^	0.267 ± 0.110	0.339 ± 0.088	0.061
Parafoveal VD of SVC (%)	39.26 ± 3.54	45.46 ± 4.08	**< 0.001**
Parafoveal VD of DVC (%)	51.16 ± 5.08	51.57 ± 4.13	0.807
Full retinal thickness, μm	295.25 ± 27.68	311.00 ± 29.69	0.120
Inner retinal thickness, μm	107.94 ± 15.21	128.38 ± 11.06	**< 0.001**
Outer retinal thickness, μm	187.13 ± 15.25	191.13 ± 6.68	0.401

*logMAR* Logarithm of the minimum angle of resolution, *BCVA* Best corrected visual acuity, *IOP* Intraocular pressure, *FAZ* Foveal avascular zone, *VD* Vessel density, *SVC* Superior vascular complex, *DVC* Deep vascular complex

### Comparison of vessel density and retinal thickness between both eyes in patients experienced with or without IOP elevation after SO tamponade

In the SO group, nine eyes (52.9%) experienced with IOP elevation during follow up. For these 9 patients, the parafoveal VD of SVC, FRT and IRT were reduced in the affected eyes compared to contralateral healthy eyes, and the differences were all statistically significant. No statistically significant differences were found for the FAZ, parafoveal VD of DVC, or ORT between the affected and contralateral healthy eyes.

For the remaining eight patients that didn’t experience with IOP elevation, IRT was reduced in the affected eyes compared to contralateral healthy eyes with statistically significant difference. While no statistically significant differences were found for the FAZ, parafoveal VD of SVC and DVC, FRT, or ORT between the affected and contralateral healthy eyes. Results are shown in Table 4.

**Table 4:** Comparison of vessel density and retinal thickness between both eyes in patients experienced with or without intraocular pressure elevation after silicone oil tamponade

SO tamponade	Patients with IOP elevation			Patients without IOP elevation		
	Affected eye	Healthy eye	*P* (Paired-t)	Affected eye	Healthy eye	*P* (Paired-t)
logMAR BCVA at follow up, Snellen equivalent	0.48 ± 0.26	0.01 ± 0.06	**0.001**	0.50 ± 0.33	0.04 ± 0.05	**0.005**
FAZ, mm^2^	0.221 ± 0.090	0.347 ± 0.100	0.052	0.319 ± 0.111	0.331 ± 0.077	0.757
Parafoveal VD of SVC (%)	38.69 ± 3.19	46.26 ± 2.32	**< 0.001**	39.91 ± 4.01	44.55 ± 5.48	0.079
Parafoveal VD of DVC (%)	50.10 ± 4.80	51.76 ± 4.95	0.482	52.34 ± 5.43	51.36 ± 3.31	0.716
Full retinal thickness, μm	290.33 ± 26.95	316.22 ± 13.08	**0.022**	305.63 ± 29.53	305.13 ± 40.24	0.976
Inner retinal thickness, μm	106.89 ± 13.66	128.89 ± 13.31	**0.013**	112.50 ± 19.02	127.88 ± 7.70	**0.025**
Outer retinal thickness, μm	183.33 ± 15.94	191.33 ± 8.82	0.286	192.88 ± 13.12	189.63 ± 4.34	0.555

*SO* Silicone oil, *IOP* Intraocular pressure, *logMAR* Logarithm of the minimum angle of resolution, *BCVA* Best corrected visual acuity, *VD* Vessel density, *SVC* Superior vascular complex, *DVC* Deep vascular complex

## Discussion

In the present study, the parafoveal VD of macular-on RRD eyes were investigated after successful retinal reattachment with a gas or SO tamponade. In the gas group, no statistically significant differences were found in FAZ, parafoveal VD of SVC and DVC between the affected eyes and the contralateral healthy eyes, in agreement with previous work [20, 21]. However, in Bonfiglio’s study, they found a lower mean DVC in the parafoveal subfield, in disagreement with our results [22]. The difference may partly come from the relatively larger RRD extension in their work (2.0 ± 0.8 quadrants). For the macular microcirculation was proved to be disturbed in macula-on RRD patients, and correlated with the extent of the retinal detachment [23]. It was possible that the baseline macular perfusion was more influenced in their cohort. For another, they followed up for at least 12 months, shorter than our 33 months. Macular microcirculation in DVC may experience recovery following successful RRD repair [24]. A longer observation period makes it easier to see the reestablishment of macular microcirculation. In the SO group, the parafoveal VD of SVC was reduced in the affected eyes compared to the healthy eyes, in agreement with previous findings by Ma [25], who postulated that maintenance of a prone position during SO tamponade may cause mechanical compression to the SVC, leading to ischemia. It is known that post-operation body position was mainly dependent on the location of the retinal breaks. The prone position was one of the primary choices [26]. Now, the body position requirements are rather flexible: patients were permitted to adjust their body based on doctors’ advice, like prone, upright, sitting or a recumbent lateral positioning, ensuring the rehabilitation first, at the same time, beneficial to decrease discomfort and increase compliance [27]. Under such circumstances, the effect of mechanical stress caused by the prone position seemed to be limited, the macula did not suffer from the buoyancy force of the SO all the time. The causes of SVC-VD changes may lie on some other aspects.

The blood supply to the superficial retina is associated with the ganglion cell complex, including the retinal nerve fiber layer (RNFL), retinal ganglion cells (RGCs), and IPL. Given the special geographic location of the SVC, a decrease in SVC-VD is more frequently discussed in the context of glaucoma. The systematic review by Flammer et al. in 2002 noted the common finding in a majority of studies that the retinal circulation was reduced in glaucomatous eyes [28]. Recently, more vascular flow details have been depicted using OCTA. The glaucomatous eyes show a lower macular VD, which preferentially affects the SVC rather than the DVC [29]. The changes related to superficial vessel perfusion that occur in glaucoma arise in part from the elevation of IOP, as confirmed by in-vivo tests [30]. The increased IOP appeared to be an unavoidable adverse event in the SO tamponade eyes [31]. Previous reports indicate that the incidence varies in a widely fluctuating range, even as high as 50%. In our study, nine eyes (52.9%) once experienced with IOP elevation during follow up, while no IOP elevation was observed in the gas group.

The IOP elevation may occur in acute or chronic form with SO tamponade. The acute manifestations, like the pupillary block or migration of SO, is prone to be found and controlled. However, the chronic IOP elevation in the mid-late phase, caused by SO emulsification, seemed to be more easily neglected. The process developed even after SO removal, which made it hard to be sensed and timely remedied [31]. The emulsification microglobules are considered to mechanically obstruct the trabecular meshwork or to be toxic to the outflow apparatus, thus inducing a rise in IOP [32]. Ultrastructural evidence has shown the presence of SO in the trabecular meshwork of enucleated eyes [33]. Immunohistochemical samples have also revealed a macrophage response associated with oil in the meshwork. Chan et al. tested the aqueous washouts after SO removal and found that more than 95% of the SO droplets in the non-clinical emulsification samples had a diameter smaller than those detectable by slit-lamp biomicroscopy (7 μm) [34].

A sub-analysis in the SO group showed that in patients suffering from IOP elevation, a lower parafoveal VD of SVC was found in the affected eyes compared with contralateral healthy eyes, while this phenomenon was not observed in patients without IOP elevation after SO tamponade. This implied that changes in IOP might participate in the process of superficial vessel perfusion loss in eyes with SO tamponade.

Besides, we observed the retinal thickness in different tamponade groups. The SO tamponade eyes showed a significantly reduced IRT compared with the contralateral healthy eyes, whereas no similar changes were detected in the gas group. The thinning of IRT, on one hand, may be induced by IOP elevation in SO tamponade eyes, as shown in glaucomatous group [35], however, in SO group sub analysis, a thinner IRT was also observed in the affected eyes of patients without IOP elevation. This indicates that some other aspects might exist to induce the changes in IRT, independent of IOP changes. The toxicity of SO to the retinal neurons might be an explanation and had been widely discussed [11, 36, 37]. The inner retina is in direct contact with SO, and experimental evidence in vitro has shown that emulsified SO can be taken up by primary retinal microglia, where it induces a pro-inflammatory response [38]. Studies with enucleated eyes applied with SO tamponade confirmed that intraretinal macrophages contained phagocytosed SO, and the eccentric pigment granules were present in the superficial retina [34]. These findings raised the speculation that a secondary inflammation related to SO itself may take part in the regional retinal injury. In our study, the affected region involves the para-fovea, a site with a high density of RGCs, which implies that RGCs are sensitive to SO contact [39]. The toxicity on the RGCs might directly influence the IRT in the SO group observed in the present study, as a result of retinal injury.

Unexplained vision loss in SO tamponade eyes after macula-on RRD surgery has been discussed in many studies [5–9]. The decreased parafoveal VD of SVC and IRT observed in the present study, which might be related to IOP elevation and SO toxicity, provides us with some ideas that might explain this phenomenon. A recent retrospective report speculated IOP elevation during SO tamponade to be the most important risk factor for vision loss [9]. In another SO tamponade cohort, 10 of 11 eyes with unexplained visual loss were found to experience with IOP elevation [8]. Similar central visual field changes have been observed in eyes with early glaucoma [40]. Furthermore, OCT examinations have presented with either a central foveal atrophy or a thickening in SO tamponade eyes, but the macular thinning was more likely to occur over long-term observation [5–8, 41]. Along with the image pixel enhancement, the thinning slabs were limited to the IRT, suggestive of injury to the innermost neuronal cells [5, 6, 8]. In consideration of these findings, the SO related perfusion-related damage and direct toxicity to macular neurons were hypothesized to lead to the subsequent vision dysfunction.

The strengths of the present study are its relatively long follow-up and its comparison with different tamponade cohorts. Only macula-sparing RRD was included in the two groups, which prevented the bias of macular flow density effects due to regional retinal detachment. However, the inherent retrospective nature and the small sample size precluded any further discovery of the potential causality in the changes in BCVA, IRT, SVC-VD, and IOP. It is more promising if we could record the dynamic changes of the indicators above during the follow-up. Further understanding of the macular visual defects occurring in SO tamponade eyes will require more macular function examinations, like microperimetry or contrast sensitivity, to identify the underlying mechanism. In addition, concerns on the optic nerve may provide new insights to explain the vision changes caused by SO.

## Conclusion

In this article, we retrospectively applied OCTA and OCT to disclose the macular vascular density and thickness details in macula-on RRD after surgery, and compared BCVA, parafoveal vessel density, and retinal thickness in gas and SO tamponade groups. Although experienced with the same surgical procedure, the SO group had a worse BCVA, lower parafoveal VD of SVC, and a thinner IRT. For this reason, the gas tamponade may be a better treatment choice in the long run for suitable patients with macula-on RRD.

## Data Availability

Most of data generated or analyzed during this study have disclosed in this article. The remaining datasets are available upon reasonable request from the corresponding author.

## Abbreviations

RRD: Rhegmatogenous retinal detachment
SO: Silicone oil
OCTA: Optical coherent tomographic angigraphy
OCT: Optical coherent tomography
SVC: Superficial vascular complex
IRT: Inner retinal thickness
